# Official definitions for undesirable medical events: Are they correctly applied in medicine?

**DOI:** 10.1007/s00508-018-1362-8

**Published:** 2018-07-12

**Authors:** Christian Smolle, Gerald Sendlhofer, Janos Cambiaso-Daniel, Michaela Sljivich, Herwig Friedl, Lars-Peter Kamolz, Gernot Brunner

**Affiliations:** 1 Research Unit for Safety in Health, Division of Plastic, Aesthetic and Reconstructive Surgery, Department of Surgery, Medical University of Graz, Auenbruggerplatz 29, 8036 Graz, Austria; 2 Division of Plastic, Aesthetic and Reconstructive Surgery, Department of Surgery, Medical University of Graz, Graz, Austria; 3 Shriners Hospital for Children—Galveston, University of Texas Medical Branch, Galveston, TX USA; 4 Department of Surgery, University of Texas Medical Branch, Galveston, TX USA; 5 School of Medicine, University of Texas Medical Branch, Galveston, TX USA; 6 Executive Department for Quality and Risk Management, University Hospital Graz, Graz, Austria; 7 Institute of Statistics, Graz University of Technology, Graz, Austria

**Keywords:** Complication, Comprehension, Medical litigation, Health literacy, Intervention

## Abstract

**Background:**

In Austria, elaborate definitions exist for the undesirable medical events side effect, adverse event, complication and medical malpractice. We aimed at investigating whether the official definitions for the abovementioned terms can be understood by a sample population representing a cross-section of the Austrian population.

**Methods:**

In this study 1021 Austrian citizens were interviewed. Demographic parameters (age, gender, occupation, level of education, monthly income, number of inhabitants at place of residence) were assessed. Participants were told the official definitions for complication, side effect, adverse event and medical malpractice and asked to select the correct definition for “complication”. The impact of sample characteristics on the ability to identify the correct definition was investigated.

**Results:**

Of the participants 315 (31%) identified the correct definition of a complication. Almost the same number (*n* = 302, 30%; χ^2^ for single samples: *p* = 0.087) falsely selected the definition for side effect. Significantly fewer (both *p* < 0.001) chose the definitions for adverse event (*n* = 220, 22%) and medical malpractice (*n* = 155, 15%). Only the respective state of origin showed significant influence on the probability of choosing the correct definition out of the four. The probability was highest in Vorarlberg (0.400) and lowest in Upper Austria (0.216, *p* < 0.001).

**Conclusion:**

For the majority the present official definitions for undesirable medical events are too complex to understand. Simple definitions for undesirable medical events should be included into patient education.

## Introduction

Medical litigation following especially surgical treatment is ever more becoming an issue if the course of the disease deviates from the expected one [1]. In a report published previously we investigated the general population’s attitude towards public healthcare in a cross-sectional telephone survey interviewing participants in Austria. More than half (54%) of all respondents stated they would issue a formal complaint if they suspected that their medical treatment might have gone wrong [2].

Possible undesirable events are discussed during any patient education, using also elaborate forms to provide patients with comprehensive information. In addition, the propagation of medical issues, in all types of media, provides information to the public at a low threshold, resulting in a basic medical literacy. Nevertheless, the fact that patients may struggle with medical information considered as “simple” [3–5] is often missed. This is why terms describing different types of untoward development during treatment of a disease, such as “side effect”, “adverse event”, “complication” and “medical malpractice” are hardly ever explained.

The aim of the present study was to investigate whether the official definitions for the abovementioned terms can be understood by a sample population representing a cross-section of the Austrian population.

## Methods

With the aid of The Austrian Gallup Institute (*Das Österreichische Gallup-Institut*), a telephone survey among Austrian inhabitants was carried out between 8 and 22 October 2015. A sample of 1021 participants above 14 years of age was drawn randomly from all 9 states in Austria. Citizens were called on their private telephones (mobile phones) and informed consent was obtained by asking whether they would agree to participate in the survey. In order to determine quality responses, careful considerations were made to ensure that interviewees were questioned during a leisure moment, a calm environment and non-distracting setting, as opposed to the middle of a work day or while driving. If necessary, the questions were repeated.

### Survey

Demographic parameters were assessed including age, gender, number of inhabitants at place of residence, occupation, highest level of education, and monthly income. In four of the nine states of Austria there is a university hospital. We assessed whether citizens lived in a state with a university hospital or not. In the questionnaire four statements were listed one of which was the correct definition of a medical complication, while the others were the correct definitions of side effect, adverse event or medical malpractice. All definitions were taken from the official gazette on the Austrian patient safety strategy published by the Austrian Ministry of Health in 2013 [5]. To eliminate possible observer bias and to obtain consistent results, the key question was asked in two different modes: first, participants were individually read each of the definitions and asked to state for each definition, whether or not it defined the term “complication” correctly. Possible answers were “yes”, “no” and “prefer not to say” (the answer “yes” could be given more than once). After completing the first portion of the interview, participants had to state which of the four previously mentioned statements, in their opinion, best defined a medical complication. Definitions were given in random order. In order to analyze the data, definitions were ranked from the most frequently selected option (a) to the least frequently selected (d) for easier comprehensibility. Table 1 shows the questionnaire and the two consequent different question modes.

**Table 1:** Questionnaire

**Part 1**
*Is the following statement the correct definition of a complication?* Possible answers for each item: yes/no/prefer not to say
(a) A complication is the worsening of a medical condition or an unexpected difficulty of a surgical intervention
*Answer:*
(b) A complication is the unintended consequence of an intervention that may also occur under sufficient accuracy
*Answer:*
(c) A complication is the consequence of an intervention that possibly, but not necessarily, causes secondary damage to the patient
*Answer:*
(d) A complication is the consequence of an intervention in which adequate accuracy was disregarded
*Answer:*
**Part 2**
*Which of the four definitions above best defines a complication?* Possible answer: a, b, c, d, prefer not to say (select only one option)
*Answer* *:*

*(a)* complication, *(b)* side effect, *(c)* adverse event, *(d)* medical malpractice

### Statistical analysis

Statistical tests were done with SPSS 23.0 for Windows (IBM Cooperation, Armonk, NY, USA) as well as R for Windows. For independent samples the χ^2^-test was applied. To evaluate the impact of demographic parameters on the answer pattern within the second question mode, a loglinear multinomial model was formed and significance of factors was determined by stepwise backwards regression analysis. The option “prefer not to say” was excluded from the analysis if present. A *p* < 0.05 was considered as statistically significant.

### Ethics

In Austria, not all surveys require an ethical approval by an institutional review board as it depends on the topic. Therefore, for this health literacy survey no ethical approval has been acquisitioned.

## Results

### Sample characteristics

Of the participants 48% were female and the most common age group was the one encompassing those between 31 and 50 years old. The majority (62%) of participants lived in places with less than 50,000 inhabitants. Employees (32%) and retired persons (24%) were the most common occupations. The most frequently reported highest level of education was a technical school degree (47%). Most participants (60%) stated to earn more than 1500 € per month, while 22% did not specify their monthly earnings and 51% lived in a state with a university hospital. For sample characteristics see Table 2.

**Table 2:** Demographic characteristics of survey participants

Parameter	*n*	%
Gender		
Female	534	52
Male	487	48
Age		
14–30 years	234	23
31–50 years	404	40
>50 years	383	37
City of residence		
<2000 inhabitants	196	19
<5000 inhabitants	235	23
<50,000 inhabitants	204	20
>50,000 inhabitants	166	16
Capital city^a^	220	22
Occupation		
Self-imployed/leading postion	160	16
Employee	329	32
Worker	149	15
Student (school/university)	70	6
Housewife	72	7
Retired	241	24
Highest level of education		
Compulsory school	267	26
Technical school	481	47
Higher school certificate/university	273	27
Declared net income (per month)		
<1500 €	182	18
1500–2400 €	215	21
2400–3000 €	125	12
>3000 €	271	27
Prefer not to say	228	22
State		
Vienna	220	22
Lower Austria	189	18
Burgenland	30	3
Styria	161	16
Carinthia	81	8
Upper Austria	180	17
Salzburg	58	6
Tirol	77	8
Vorarlberg	25	2

^a^Vienna: 1,840,573 inhabitants (updated 1 January 2016)

### Comprehension of the term “complication”

**Fig. 1:**
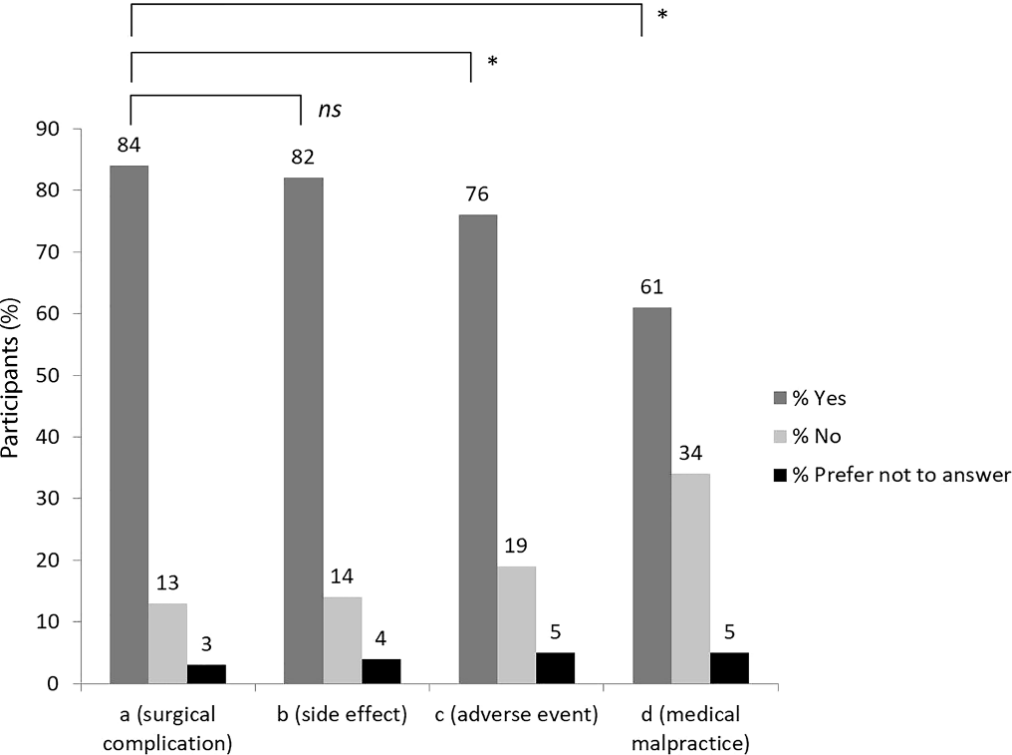
Results obtained from survey participants for comprehension of the term “complication”. (*ns* not significant, * *p* < 0.001)

Of the participants 857 (84%) correctly deemed option “a” to be the correct definition of a complication. Significantly fewer participants presumed options “c” (adverse event, *n* = 776, 76%) or “d” (medical malpractice, *n* = 627, 61%) as the correct definition of a complication (χ^2^-test, both *p* < 0.001). In comparison to the correct definition of a complication (“a”), option “b” (side effect, *n* = 838, 82%) was deemed the correct answer almost as often (χ^2^-test, *p* = 0.295; not significant). Fig. 1 shows results from the first part of the questionnaire.

### Ability to select the correct definition for a complication out of four

When participants were asked to select the correct term for “complication” out of the abovementioned four official definitions, 315 (31%) chose option “a”, 302 (30%) chose “b”, 220 (22%) chose “c”, 155 (15%) selected option “d” and 29 (3%) preferred not to answer that question. Compared to the chance probability of choosing the right definition (25%), the number of participants selecting the correct definition (“a”) of a complication was higher (χ^2^-test, *p* < 0.001). The definitions of medical malpractice and adverse event were chosen less often than the one for complication (χ^2^, both *p* < 0.001). The numbers of participants erroneously choosing the definition of a side effect (option “b”) and of those who selected the correct definition (option “a”) did not differ significantly (χ^2^, *p* = 0.087).

### Factors associated with the probability of choosing the correct definition for “complication”

**Fig. 2:**
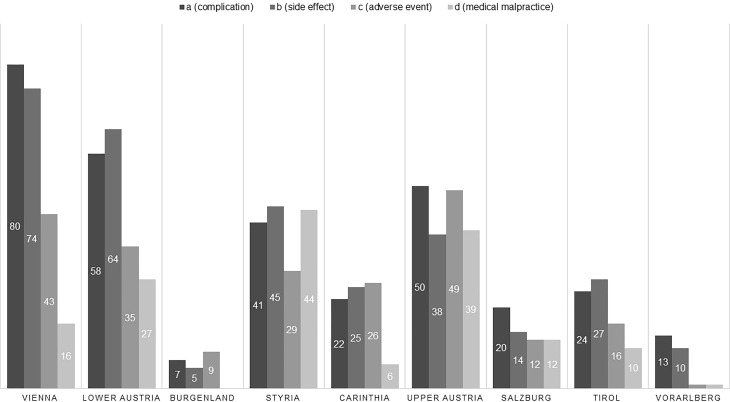
Distribution of answers in the second question mode depending on the federal state (absolute values)

In the loglinear multinomial regression model, only the respective state of origin had a significant influence on the probability of choosing a specific answer (*p* < 0.001). The probability for choosing the correct definition for complication was highest in Vorarlberg (0.5200) and lowest in Styria (0.258). Vice versa the probability of erroneously choosing the definition for medical malpractice was lowest for participants from Burgenland (0.000) and highest for participants from Styria (0.277). Detailed results are listed in Table 3 while Fig. 2 shows the distribution of answers in the different federal states.

**Table 3:** Analysis of demographic factors regarding the probability of choosing the correct definition of a complication

State	Probability of answer “a” (complication; correct)	Probability of answer “b” (side effect)	Probability of answer “c” (adverse event)	Probability of answer “d” (medical malpractice)
Vienna	0.376	0.347	0.202	0.075
Lower Austria	0.315	0.348	0.190	0.147
Burgenland	0.333	0.238	0.429	0.000
Styria	0.258	0.283	0.182	0.277
Carinthia	0.278	0.316	0.329	0.077
Upper Austria	0.284	0.216	0.278	0.222
Salzburg	0.345	0.241	0.207	0.207
Tirol	0.312	0.351	0.208	0.129
Vorarlberg	0.520	0.400	0.040	0.040

## Discussion

Several investigatory reports have revealed that patients may not understand or are even unable to retain medical information. These facts must be taken into consideration when explaining medical procedures, especially if informed consent is required [3, 6–8]. Our survey revealed two major findings: firstly, our results suggest that the general population has difficulty defining the term complication correctly with the given official definitions. Of all participants, only 31% picked the correct definition of complication when confronted with four official descriptions for different medical events. Although this number is significantly greater than the chance probability of making the correct choice (25%), it is necessary to keep in mind that vice versa 69% failed to define “complication” correctly. Also, the ability to discriminate between the definitions for “side effect” and “complication” was poor in the present collective.

The second and probably even more concerning finding was that depending on the question mode 61% and 15%, respectively, of the respondents defined a complication with the sentence “a complication is the consequence of an intervention in which adequate accuracy was disregarded”. Although this option was chosen by the fewest number of participants in both cases, this actually means that almost two out of three patients may regard a complication as the physician’s fault. Furthermore, it can be assumed that one in seven patients is absolutely convinced a complication is the doctor’s fault, as reflected by the 15% selecting the definition for malpractice when asked to choose the most suitable definition for a complication out of the four. This finding was even more pronounced in certain federal states, e. g. Styria, where the probability of participants selecting the definition for medical malpractice was as high as 28%, or Upper Austria where the probability was 22%. Taken into account that up to 95% of surgical patients are concerned about perioperative complications [9], our findings are even more striking. Poor comprehension alone cannot explain why one in seven participants in our study placed the term “complication” on an equivocal level with the definition for “medical malpractice”. In fact, that very definition was the only one unambiguously insinuating that a complication was the caregiver’s fault. Probably the abilities of modern healthcare are overestimated and unfortunate events are not taken into account by the general public. This should be taken into consideration when patients are educated about their treatment options and accompanying risks.

According to Metcalfe et al. unexpected misunderstandings emerging from informed consent also constitute a major reason for medical litigation [1]. Comprehension of medical information is commonly referred to as health literacy. Several studies have investigated health literacy among different patient groups. Altogether, better comprehension and recall of medical information was frequently associated with higher levels of education [3, 4, 6, 10–12], higher income [3, 11, 12] and younger age [3, 4, 6, 11, 12]. In our study, however, neither the level of education nor age had a significant impact on the correct definition of the term “complication”.

Although women have been seen to have better medical knowledge and higher health awareness than men [13], the ability of both men and women in our study to identify the correct wording for “complication” was almost identical. The medical literature is inconsistent concerning the observation that health literacy is better in urban populations [14, 15]. In the present analysis a better recognition of the correct definition was also not evident among participants from more populated areas; however, answer patterns differed between the federal states. In our study recognition of the correct definition for the term “complication” was good among participants from Vorarlberg, Vienna and Salzburg, whereas it was bad among participants from Styria, Upper Austria and Carinthia. This is to certain extent comparable to the findings of the nationwide health study from 2012 by Pelikan et al. who found highest levels of adequate or excellent health literacy in Burgenland and Vorarlberg and lowest levels in Styria and also Vienna [16].

Considering the meaning of a side effect and that of a complication to a patient, a side effect is often considered as a circumstance occurring during therapy that is “common”, “normal” or even “to be expected” in a certain proportion of patients. A complication is however considered as “uncommon”, “error” or even “failure of therapy” and negatively affects the patient’s perception of the quality of delivered care [17–19]. Nevertheless, discrimination between the two definitions was poor among the present study population.

Finally, the knowledge about the differences between “side effect”, “adverse event”, “complication” and “medical malpractice” seems to be taken for granted, which is why the terms are neither elucidated in the media nor in medical everyday routine. Thus, the complexity of the definitions used in our telephone survey may have overstrained a part of the study population. Although obviously correct from the lawgivers’ point of view the terms are difficult to grasp by people not familiar with juridical expressions. This hypothesis is further strengthened by the fact that none of the usual health literacy-associated factors (young age, higher income, better education, high job position) had an impact on recognizing the correct definition of a medical complication.

Our study has several limitations: firstly, the participants were recruited solely from Austria. Thus, the collective may not be representative for populations outside German speaking countries due to legislative and sociocultural differences and differences in the delivery of medical care. Secondly, the fact that the survey was carried out via telephone may have increased the difficulty of the task of identifying the correct definition for a complication. Lastly, a non-patient population was interviewed. It cannot be ruled out that our results may not be applicable to hospitalized patients.

To conclude, patients need better information on undesirable medical events. This aspect should be included into patient education; however, in this respect the present official definitions are probably inappropriate, since they are difficult to understand, making even well-educated people struggle. Simple definitions for medical events would be desirable.
